# Trends in Tanning Bed Use, Motivation, and Risk Awareness in Germany: Findings from Four Waves of the National Cancer Aid Monitoring (NCAM)

**DOI:** 10.3390/ijerph16203913

**Published:** 2019-10-15

**Authors:** Katharina Diehl, Tatiana Görig, Rüdiger Greinert, Eckhard W. Breitbart, Sven Schneider

**Affiliations:** 1 Mannheim Institute of Public Health, Social and Preventive Medicine, Medical Faculty Mannheim, Heidelberg University, Ludolf-Krehl-Str. 7-11, 68167 Mannheim, Germany; tatiana.goerig@medma.uni-heidelberg.de (T.G.);; 2 Arbeitsgemeinschaft Dermatologische Prävention (ADP) e. V., Cremon 11, 20457 Hamburg, Germany; grein25@gmx.de (R.G.); info@professor-breitbart.de (E.W.B.)

**Keywords:** tanning bed use, artificial tanning, skin cancer, melanoma, indoor tanning

## Abstract

Indoor tanning is an important risk factor for the development of melanoma and non-melanoma skin cancer. With our nationally representative monitoring, we aimed at describing tanning bed use, user characteristics, reasons for use, and risk awareness over time. In the framework of the National Cancer Aid Monitoring (NCAM), we collected representative data on 12,000 individuals aged 14 to 45 years in annual waves of n = 3,000 participants in Germany between 2015 and 2018. We used descriptive statistics and chi²-tests to uncover group differences. To compare data from the different waves, we calculated confidence intervals. The use of tanning beds decreased from 2015 (11.0%, 95%-CI: 9.9%–12.1%) to 2018 (8.8%, 95%-CI: 7.8%–9.8%). However, this decrease did not affect all subgroups. For instance, there was an (non-significant) increase in minors and the prevalence remained stable for individuals with immigrant background and males. Attractiveness was an important reason for tanning bed use in each wave. Over time, there was an increase in medical-related reasons for use. Furthermore, monitoring showed a decrease in risk awareness regarding tanning bed use and ultraviolet (UV) radiation. While it is a positive development that the overall use of tanning beds in Germany has decreased over time, the increasing use by minors despite the legal ban is alarming. Due to the declining risk awareness it is necessary to implement prevention and education campaigns specifically targeted at this group.

## 1. Introduction

Indoor tanning is a well-known risk factor for the development of melanoma and non-melanoma skin cancer [1]. In 2009, it was classified as carcinogenic to humans by the International Agency for Research on Cancer (IARC) [1]. In a review and meta-analysis, Boniol et al. [2] calculated that, in Europe, 3,438 of 63,942 new cases of melanoma would be related to tanning bed use. The ever use of a tanning bed increased the risk of melanoma by 20%; each additional tanning session per year increased the risk by 1.8% [2]. Since the incidence of skin cancer increases worldwide [3], further prevention measures need to be implemented to decrease such an avoidable risk factor. For the successful development of comprehensive prevention strategies, it is crucial to collect as much detailed and relevant data as possible on user characteristics, reasons for use, and risk awareness related to use of tanning beds as well as the development of tanning bed use over time.

Numerous studies have been conducted on tanning bed use in various countries, predominantly in the United States, to gather trend data. The focus of research was mainly on adolescents [4,5,6,7,8,9,10,11,12] and often the change in tanning bed use prior to and after legislation regarding minors was quantified [10,12]. Most data monitoring tanning bed use are derived from large representative studies in which tanning bed use was only assessed on the basis of a few questions [4,5,6,7,9,10,13]. Other studies were not representative [8]. Most assessments were not conducted annually or biannually and years elapsed between the individual data collections [8,11,12].

However, there are also studies that stand out in this area of research: First, there is a long-term data analysis from Norway based on various data sources, including the Norwegian Sunbed Association, which provides data from 1980 to 2011 [14]. Second, several sun surveys were conducted annually in Denmark between 2007 and 2015 [15,16,17]. Initially this Danish study was designed as a tool to monitor the success of the sun safety campaign; thus, due to the high awareness of the topic, results may be biased as indicated by the authors [16]. Third, in 2011 and 2013, a tracking survey on tanning bed use was part of the British SunSmart Campaign [18].

While these trend analyses gave a good overview of the development in tanning bed use in the respective country, they did not deliver more detailed information on reasons for tanning bed use and risk awareness. As information on these behavioral factors associated with tanning bed use are important for the development of future prevention strategies, we established a unique nationwide system for long-term monitoring of tanning bed use and related aspects, funded by the German Cancer Aid. Our aim was—similar to other nationwide monitoring systems on oncological risk factors such as the consumption of tobacco and alcohol—to collect information on the development of tanning bed use within the German population. In addition, we aimed to identify potential changes in the reasons for sunbed use and risk awareness.

## 2. Materials and Methods

We used data of the first four waves (2015, 2016, 2017, 2018) of the National Cancer Aid Monitoring (NCAM) conducted by the author panel and funded by the German Cancer Aid. The study was approved by the ethical committee of the Medical Faculty Mannheim, Heidelberg University (2007-269E-MA). Four further waves will be conducted between 2019 and 2022.

### 2.1. Data Collection

Each year from October to December, data were collected using cross-sectional standardized telephone interviews among 3,000 individuals between 14 and 45 years living in Germany [19]. Interviews were conducted by trained interviewers. Sampling followed a two-stage procedure: First, telephone numbers (since 2016 cell phone numbers as well) were selected randomly, following the well-established German telephone sampling systems RTS and ADM. Second, the person in the household whose birthday is next was asked to participate in the survey. The following criteria applied for participation: age between 14 and 45 years, sufficient German language skills, and provision of informed consent. The response rates following American Association for Public Opinion Research (AAPOR) standard Respsonse Rate (RR) 3 were 32.1% (2015), 27.9% (2016), 29.2% (2017), and 28.5% (2018). Data are weighted by sex, age, education, and state of residence to be representative for Germany.

### 2.2. Measurement Tools

Our main outcome variable is current tanning bed use, which is defined as at least one tanning session within the last 12 months. Furthermore, we asked for the number of tanning sessions within the last 12 months to be able to calculate mean and median. Further questions included the location of the last tanning session (categorized as tanning salon vs. other facilities) and the use of goggles during the last visit (yes vs. no).

As independent variables to describe current tanning bed use in more detail, we included sex (female vs. male), age (14–17 years vs. 18–25 years vs. 26–35 years vs. 36–45 years), immigrant background (calculated following an established German assessment [20], which results in a binary variable: yes vs. no), occupational status (no occupation vs. part-time vs. full-time), and school education (low vs. medium vs. high).

Additionally, we asked for potential reasons for use of tanning beds in all four waves (only for participants who ever used a tanning bed). The following reasons were included: relaxation, enhancement of attractiveness, pre-tanning for holiday, light and warmth, vitamin D supplementation, health care, skin diseases, and physician’s recommendation. Respondents could either agree or disagree with each of the items.

Furthermore, risk awareness of tanning beds and UVR was assessed by the following three statements: “Each incidence of sunburn leaves permanent damage in the skin”, “Regular use of tanning beds causes premature skin aging”, and “Regular use of tanning beds causes skin cancer”. For each statement, respondents could choose between the answer categories “rather agree”, “rather disagree”, and “don’t know”.

### 2.3. Statistics

Besides descriptive analyses, we calculated chi²-tests to identify potential statistic significant differences between subgroups. A *p*-value < 0.05 was considered significant. All analyses were conducted with IBM SPSS Statistics Version 25 (IBM, Armonk, NY, USA).

## 3. Results

In each wave, 3,000 individuals between 14 and 45 years of age participated in the telephone interviews. Data are weighted based on the German Microcensus to make the study more representative for Germany. A detailed description of the study population for each wave can be found in Table 1.

**Figure 1:**
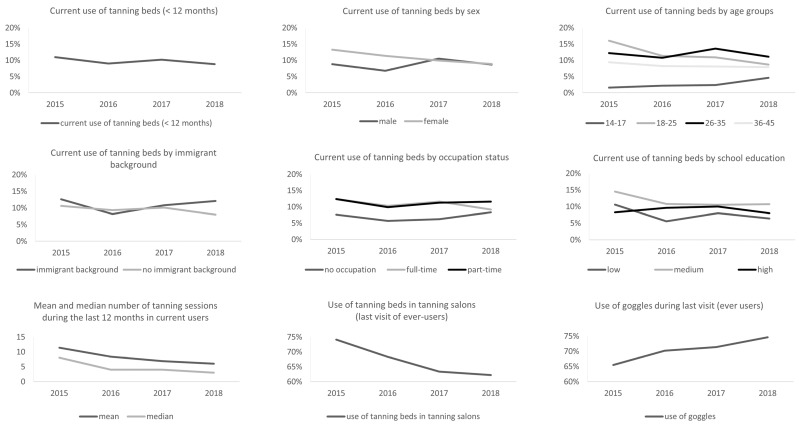
Development of tanning bed use from 2015 to 2018 in Germany. Data are based on the waves 2015, 2016, 2017, and 2018 of the National Cancer Aid Monitoring (NCAM). Data are weighted by sex, age, and education to be representative for Germany. n = 3000 individuals aged 14 to 45 years (per wave).

The current use of tanning beds decreased significantly from 2015 (11.0% (9.9%–12.1%)) until 2018 (8.8% (7.8%–9.8%), Figure 1). This decrease applies for females, where we found a continuous decrease from 13.3% (11.6%–15.0%) in 2015, to 11.4% (9.8%–13.0%) in 2016, 9.9% (8.4%–11.4%) in 2017 and 8.9% (7.4%–10.4%) in 2018. While we found significant differences between females and males in 2015 and 2016 (both *p* < 0.001), this lower tanning bed use in males levels off at the prevalence in females. Overall, the prevalence of males remained stable from 2015 (8.8% (7.4%–10.2%)) to 2018 (8.7% (7.3%–10.1%)).

Regarding age, we see a (significant) decrease in current tanning bed use over time in the age groups 18–25 years (16.0% (13.3%–18.7%) in 2015 to 8.7% (6.6%–10.8%) in 2018), 26–35 years (12.2% (10.2%–14.2%) in 2015 to 11.1% (9.2%–13.0%) in 2018), and 36–45 years (9.4% (7.6%–11.2%) in 2015 to 7.9% (6.2%–9.6%) in 2018). However, there was an increase of tanning bed use in the age group 14–17 years (1.6% (0.2%–3.0%) in 2015 to 4.6% (2.2%–7.0%) in 2018).

Over time, we see no significant decrease for respondents with immigrant background, while we see a decrease in respondents without immigrant background from 10.6% (9.4%–11.8%) in 2015 to 8.0% (6.9%–9.1%) in 2018. The prevalence in 2018 differed significantly between those with immigrant background (12.1%) and those without (8.0%; *p* = 0.002). Regarding occupation, we did not find any significant changes over time. However, in waves 2015 (*p* = 0.001), 2016 (*p* = 0.001) and 2017 (*p* < 0.001), we found significant differences between the groups with the lowest prevalence in those without occupation.

Regarding school education, we found significant differences between low, medium, and highly educated individuals in 2015 (*p* < 0.001), 2016 (*p* = 0.002), and 2018 (*p* = 0.006) with the highest prevalence in those with medium education. When we compare waves 2015 and 2018, we see a decrease in current tanning bed use in all three groups. The only statistically significant change was found for low educated respondents (2015: 10.6% (8.3%–12.9%); 2018: 6.4% (4.6%–8.2%)).

In current users, the number of tanning sessions during the last 12 months decreased from 11.4 (10.3–12.5) (median: 8 (6.9–9.1)) to 6.0 (5.2-6.8) (median: 3 (2.2–3.8)). We found a significant decrease in use of tanning beds in tanning salons from 74.1% (71.2%–77.0%) in 2015 to 62.2% (58.9%–65.5%) in 2018. In the same time, the use of goggles during the tanning session increased from 65.5% (62.3%–68.7%) to 74.6% (71.6%–77.6%). However, there was no clear trend in the provision of goggles by the provider of the tanning bed (data not shown).

**Figure 2:**
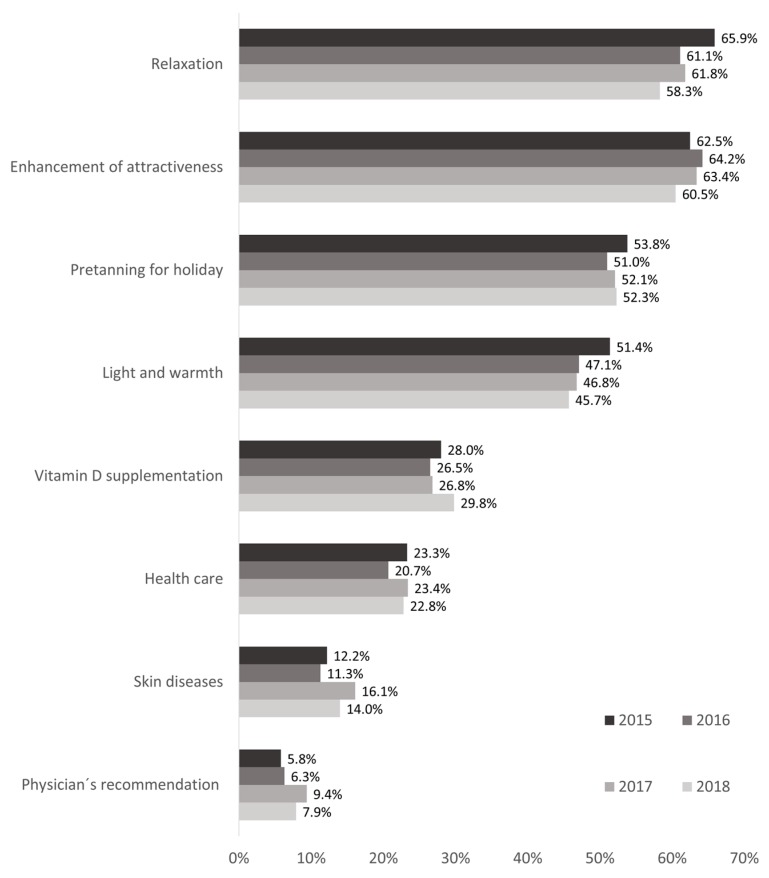
Reasons for tanning bed use from 2015 to 2018. Data are based on the waves 2015, 2016, 2017, and 2018 of the National Cancer Aid Monitoring (NCAM). Data are weighted by sex, age, and education to be representative for Germany. Shown are data on ever users aged 14 to 45 years.

Regarding reasons for tanning bed use, we see a significant decrease from 2015 (65.9% (62.8%–69.0%)) to 2018 (58.3% (54.9%–61.7%); Figure 2) for relaxation. In addition, we found a decrease for feeling of light and warmth (n.s.), while reasons such as enhancement of attractiveness and pre-tanning for holiday remain quite stable. The four medical-related reasons showed mixed results: while health care as a reason for tanning bed use revealed no significant changes, we see an increase in the importance of vitamin D supplementation (n.s.), skin diseases (significant change from 2016 to 2017), and physician’s recommendation (significant change from 2015 to 2017).

Comparing the reasons for use in current and past users of tanning beds, we found significant differences for some reasons in several waves: While relaxation was important for much more current users than past users in 2015 (75.2% vs. 60.9%, *p* < 0.001), this turned around in 2018, where 51.1% of current users and 61.6% of past users indicated this as a reason for use (*p* = 0.013). The same applied for feeling of light and warmth, which was a reason for more current than past users in 2015 (63.3% vs. 44.0%, *p* < 0.001) and 2016 (52.0% vs. 44.6%, *p* = 0.029), but for less current than past users in 2018 (38.4% vs. 49.3%, *p* = 0.010). Enhancement of attractiveness was rather a reason for current users than past users (2015: *p* < 0.001; 2016: *p* = 0.003; 2018: *p* = 0.025). While health care as a reason for tanning bed use revealed significant differences in 2015 (current: 30.0% vs. past: 19.2%; *p* < 0.001) and 2016 (current: 28.4% vs. 16.4%; *p* < 0.001), there were no significant differences in the subsequent waves. 

Pre-tanning for holidays and physician’s recommendation showed significant differences between current and past users in 2015; vitamin D supplementation was named as a reason by more current than past users in 2018 (34.1% vs. 27.9%, *p* = 0.012).

**Figure 3:**
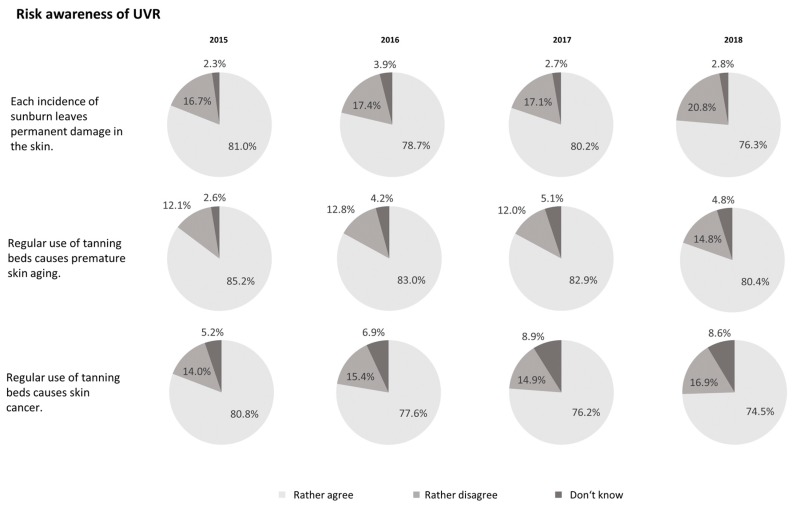
Risk awareness regarding ultraviolet radiation and tanning bed use from 2015 to 2018. Data are based on the waves 2015, 2016, 2017, and 2018 of the National Cancer Aid Monitoring (NCAM). Data are weighted by sex, age, and education to be representative for Germany. n = 3000 individuals aged 14 to 45 years (per wave).

Risk awareness of UV radiation and tanning bed use decreased significantly over time (Figure 3). Less individuals agreed that each incidence of a sunburn leaves permanent damage in the skin in 2018 (76.3% (74.8%–77.8%)) compared with 2015 (81.0% (79.6%–82.4%)). The same applied for “Regular use of tanning beds causes premature skin aging” (80.4% (79.0%–81.8%) in 2018 vs. 85.2% (83.9%–86.5%) in 2015) and “Regular use of tanning beds causes skin cancer” (74.5% (72.9%–76.1%) in 2018 vs. 80.8% (79.4%–82.2%) in 2015).

Subgroup analysis on risk awareness by tanning bed use showed significant differences for each statement in each wave: current users of tanning beds were less likely to agree to the above-named statements compared to past and never users. Comparing data from 2015 and 2018, there is a decrease in risk awareness for all three items in all three groups. The only exception is the item on skin cancer risk, for which current users show an increase (2015: 68.3%; 2018: 72.9%). However, they remain the group with the lowest agreement. Decrease regarding risk awareness was up to 12.4 percentage points (i.e., agreement to the item on sunburn among current tanning bed users: 77.0% vs. 64.6%).

## 4. Discussion

With the first four waves of the nationally representative NCAM, we had the unique opportunity to monitor the prevalence of current tanning bed use, the development of motives for tanning bed use, and the development of risk awareness over a period of four years. We found a decrease in current use of tanning beds, even though this decrease did not affect all subgroups. Furthermore, we revealed a decreasing risk awareness regarding potential consequences of UV radiation and tanning bed use.

### 4.1. Use of Tanning Beds

Fortunately, there is a decrease in the prevalence of tanning bed use, which was also shown in other populations over time [4,6,10,13,16,17]. Besides the general decrease in tanning bed use, the number of tanning sessions in current users also declined. This may indicate at least some success of prevention measures. Still, we identified subgroups in which this decrease was not reflected: men, minors, and individuals with immigrant backgrounds.

While we identified a significant decrease in tanning bed use among females, which was also found for other countries [4,5,7,9,13,16,17], this was not the case for males. These results are in line with several previous studies [4,5,7,13], while other studies reported a decrease for men as well [9,10,16,17]. Both sexes showed a comparable prevalence in 2018 (females: 8.9%; males: 8.7%), although there was a difference of 4.5 percentage points in 2015 (females: 13.3%; males: 8.8%). One reason supporting these figures might be the overall tendency of men to take more risks and to be less risk-aware, while females are more susceptible to health promotion campaigns, warning against tanning bed use. A representative study of the authors in 2011/2012 showed that females are more risk aware regarding the potential dangers of UV radiation and tanning bed use [21]. Following models on behavioral change, this knowledge may influence attitude and behavior.

Regarding age, we found a decrease in tanning bed use in the age groups of 18 to 45 years [13]. Unfortunately, there has been an increase in the age group of 14 to 17 years, despite the introduction of a ban on the use of tanning beds for minors in Germany in 2009. Minors seem to find ways around the ban and get access to tanning beds. These findings underline the necessity for tighter controls [22].

The decrease in current tanning bed use was found in all educational groups. However, we found differences regarding immigrant background: While prevalence decreased in individuals without immigrant background, it remained stable in those with immigrant background. Here, different explanations may be possible: differences in beauty ideal [23], differences in their skin type (e.g., a higher proportion of those with skin type III and higher in individuals with immigrant background), higher risky sun exposure behavior in individuals with immigrant background (i.e., higher likelihood of sunbathing and lower likelihood of using sun protection measures [24]) and differences in susceptibility to health promotion (e.g., different health-related habits and lower participation in prevention programs [25]).

### 4.2. Reasons for Tanning Bed Use

Reasons such as relaxation and feeling of light and warmth became less important over the course of the four waves of monitoring. Enhancing attractiveness as a motive for using tanning beds remained stable and played an important role, especially for current users. This corresponds with literature on reasons for tanning in general: Tanned skin is perceived as being attractive, beautiful, and healthy [26,27].

In addition, we see an increase in importance for medical-related reasons: vitamin D supplementation, skin diseases, and physician’s recommendation. The trend for vitamin D supplementation as reason for use is especially prevalent amongst current users. These findings suggest that the industry’s typical advertising messages find a ready market in the target group. However, while tanning beds may be a source for vitamin D, the WHO strongly advises against using tanning beds [28]. Following the WHO [28], potential risks of tanning beds overweigh potential benefits.

### 4.3. Risk Awareness

Findings on risk awareness regarding tanning bed use and UV radiation are alarming, because it decreased over time. Users of tanning beds, especially, showed a lower awareness of potential risks compared to past and never users. This underlines the need for target group specific education and prevention [21].

### 4.4. Potential Limitations of the Study

Our monitoring builds an important base for future (targeted) prevention—to warn against the potential negative consequences of tanning bed use—and for structural or regulatory interventions. When interpreting our findings, some potential limitations should be considered. A first limitation might be the focus on the age group 14- to 45-year olds. To eliminate the problem, we will include 16- to 65-year olds in the following four waves (2019, 2020, 2021, 2022). This enables a more widespread inclusion of the population. Second, our data are based on self-reports. Therefore, we cannot exclude a bias regarding recall and social desirability. A detailed pretest on test-retest-reliability showed that a recall bias can be neglected [23]. Since tanning bed use is—at least in Germany—not seen as a socially undesired behavior, such as smoking, we assume that the respondents answered truthfully. Our cognitive pretests did not yield critical information.

### 4.5. Implications of Study Findings for Prevention

Although the overall prevalence of tanning bed use is decreasing, there are subgroups that still show a stable prevalence, or even an increase. This calls for targeted prevention and education, for instance in adolescents, who showed an increase in tanning bed use prevalence over time. Here, education in schools about the health consequences of UV radiation may be helpful; stricter control in tanning studios and other facilities is also vital. Since Germany has had a tanning bed use ban for minors since 2009 [22], no minor should be able to use a public tanning bed. In addition, a ban on unsupervised tanning beds in swimming pools or fitness studios, as in other countries, may be effective.

In Germany, there was a lot of reporting in the media between 2009 and 2012 in the course of the introduction of legislation on tanning bed use. However, nowadays in Germany, there is not a big focus in prevention on the use of tanning beds. It is thus important to increase public awareness on the potential health risks of tanning bed use. Apart from health-related messages, discussions on attractiveness might also be successful: excessive UV exposure is regarded as a central risk factor for local skin alterations, such as pigmentation disorders (age spots, melisma [29]) and premature skin aging, which are rather relevant to aesthetics than to health. However, in Germany, as in other Western countries, there is a strong tie between tanned skin and attractiveness, which is also reflected in the reasons for tanning bed use. This reveals a paradox. In Asian countries, for instance, pale skin is perceived as attractive with a trend to brighten the skin. However, a lot of time is needed to counteract Western cultural beauty ideals. Here, social media could be helpful, especially in changing beauty ideals in young people and promoting pale skin as being attractive.

## 5. Conclusions

The current use of tanning beds is fortunately decreasing in Germany. However, there are subgroups such as men and minors, in which we do not see any changes and an increase, respectively. In addition, the increase in tanning bed use for medical reasons calls for further education, as their risks outweigh potential health benefits. Furthermore, the decrease in risk awareness—especially among current users of tanning beds—calls for targeted prevention. Since NCAM funding was prolonged by four additional waves, we will be able to monitor the future development of tanning bed use, reasons for use, and risk awareness.

## Figures and Tables

**Table 1:** Sociodemographic characteristics of study participants in waves 2015 to 2018.

Sociodemographic Characteristics		2015	2016	2017	2018
Women		49.1%	48.6%	48.5%	48.6%
Age					
	14–17 years	10.6%	12.2%	8.5%	10.2%
	18–25 years	22.9%	21.3%	24.8%	23.0%
	26–35 years	33.7%	34.3%	34.5%	34.5%
	36–45 years	32.8%	32.2%	32.2%	32.3%
Immigrant background		19.4%	17.2%	18.9%	19.2%
With a partner		62.0%	63.9%	63.7%	62.0%
Education level					
	Low	25.3%	22.8%	26.5%	25.8%
	Medium	37.2%	38.4%	36.6%	36.9%
	High	37.5%	38.8%	36.9%	37.3%
Occupation					
	full-time	52.0%	55.6%	54.2%	56.0%
	part-time	20.2%	20.1%	21.5%	23.7%
	None	27.8%	24.3%	24.3%	20.3%

Data are based on the waves 2015, 2016, 2017, and 2018 of the National Cancer Aid Monitoring (NCAM). Data are weighted by sex, age, and education to get as representative as possible for Germany. n = 3000 individuals aged 14 to 45 years (per wave).
